# Distinct Brain Regions in Physiological and Pathological Brain Aging

**DOI:** 10.3389/fnagi.2019.00147

**Published:** 2019-06-18

**Authors:** Jin San Lee, Yu Hyun Park, Seongbeom Park, Uicheul Yoon, Yeongsim Choe, Bo Kyoung Cheon, Alice Hahn, Soo Hyun Cho, Seung Joo Kim, Jun Pyo Kim, Young Hee Jung, Key-Chung Park, Hee Jin Kim, Hyemin Jang, Duk L. Na, Sang Won Seo

**Affiliations:** ^1^Department of Neurology, Samsung Medical Center, Sungkyunkwan University School of Medicine, Seoul, South Korea; ^2^Neuroscience Center, Samsung Medical Center, Seoul, South Korea; ^3^Department of Neurology, Kyung Hee University Hospital, Seoul, South Korea; ^4^Department of Biomedical Engineering, Daegu Catholic University, Gyeongsan, South Korea; ^5^Department of Neurology, Chonnam National University Medical School, Gwangju, South Korea; ^6^Department of Neurology, Gyeongsang National University School of Medicine and Gyeongsang National University Changwon Hospital, Changwon, South Korea; ^7^Samsung Alzheimer Research Center, Center for Clinical Epidemiology, Samsung Medical Center, Seoul, South Korea; ^8^Department of Health Sciences and Technology, Clinical Research Design and Evaluation, SAIHST, Sungkyunkwan University, Seoul, South Korea

**Keywords:** physiological brain aging, pathological brain aging, cortical thickness, Alzheimer’s disease, precuneus, inferior temporal region

## Abstract

**Background:**

Studying structural brain aging is important to understand age-related pathologies, as well as to identify the early manifestations of the Alzheimer’s disease (AD) continuum. In this study, we investigated the long-term trajectory of physiological and pathological brain aging in a large number of participants ranging from the 50s to over 80 years of age.

**Objective:**

To explore the distinct brain regions that distinguish pathological brain aging from physiological brain aging using sophisticated measurements of cortical thickness.

**Methods:**

A total of 2,823 cognitively normal (CN) individuals and 2,675 patients with AD continuum [874 with subjective memory impairment (SMI), 954 with amnestic mild cognitive impairment (aMCI), and 847 with AD dementia] who underwent a high-resolution 3.0-tesla MRI were included in this study. To investigate pathological brain aging, we further classified patients with aMCI and AD according to the severity of cognitive impairment. Cortical thickness was measured using a surface-based method. Multiple linear regression analyses were performed to evaluate age, diagnostic groups, and cortical thickness.

**Results:**

Aging extensively affected cortical thickness not only in CN individuals but also in AD continuum patients; however, the precuneus and inferior temporal regions were relatively preserved against age-related cortical thinning. Compared to CN individuals, AD continuum patients including those with SMI showed a decreased cortical thickness in the perisylvian region. However, widespread cortical thinning including the precuneus and inferior temporal regions were found from the late-stage aMCI to the moderate to severe AD. Unlike the other age groups, AD continuum patients aged over 80 years showed prominent cortical thinning in the medial temporal region with relative sparing of the precuneus.

**Conclusion:**

Our findings suggested that the precuneus and inferior temporal regions are the key regions in distinguishing between physiological and pathological brain aging. Attempts to differentiate age-related pathology from physiological brain aging at a very early stage would be important in terms of establishing new strategies for preventing accelerated pathological brain aging.

## Introduction

Aging is a physiological process that affects all tissues and organs including the human brain. The functional capabilities of the brain show a gradually progressive decline during aging, as with other organs. Specifically, declines in memory, conceptual reasoning, and processing speed are commonly observed in the elderly (Blazer et al., 2015). To date, the underlying cellular and molecular mechanisms of brain aging have been established in the context of mitochondrial dysfunction, impaired molecular waste disposal, aberrant neuronal network activity, oxidative damage, dysregulation of neuronal calcium homeostasis, and inflammation (Mattson and Arumugam, 2018).

Alzheimer’s disease (AD) is a slowly progressing, irreversible neurodegenerative disease with a long preclinical phase (Masters et al., 2015). Regarded as a pre-dementia phase of AD, amnestic mild cognitive impairment (aMCI) is characterized by the development of noticeable memory problems, which do not affect the independence of functional abilities (Albert et al., 2011). Based on the concept of the paradigm shift in focus from AD dementia to preclinical AD in clinical trials, early-stage aMCI with milder cognitive impairments has emerged as a new classification rather than late-stage aMCI (Aisen et al., 2010). Furthermore, subjective memory impairment (SMI), a self-experienced persistent memory decline without objective cognitive deterioration, may represent the first symptomatic manifestation of AD (Jessen et al., 2014; Rabin et al., 2017). Therefore, both the clinical symptomatology and the underlying process of AD pathology have been conceptualized as part of the AD continuum which includes: SMI, aMCI, and AD dementia (Sperling et al., 2011).

Studying structural brain aging is of interest for the understanding of age-related pathology, as well as for the identification of the early manifestations of the AD continuum. Indeed, a previous study from our group showed that physiological brain aging occurs from the age of 40 years and continues past the age of 80 years (Lee et al., 2018). Cortical thickness in the dorsolateral prefrontal cortex and inferior parietal lobule was affected by aging earlier in life, but cortical thickness was relatively preserved in the precuneus, inferior temporal, and lateral occipital cortices until later in life. However, neuroimaging studies of AD have shown greater cortical atrophy in the medial temporal, posterior parietotemporal, posterior cingulate, and precuneus in early stage of disease (Jack, et al., 1997; Scahill et al., 2002; Damoiseaux et al., 2012). Also, there have been reports showing that cortical atrophy precedes cognitive decline and can be used to detect early changes in AD (Hampel et al., 2008; Jack et al., 2009). Additionally, patients with aMCI exhibit significant atrophy in the hippocampus, parahippocampal gyrus, and entorhinal cortex compared to cognitively normal (CN) individuals (Killiany et al., 2000; Wolf et al., 2001). Even in SMI stage, significant cortical atrophy in the entorhinal cortex, posterior cingulate gyrus, and inferior parietal cortex is observed (Peter et al., 2014; Meiberth et al., 2015; Schultz et al., 2015). To our knowledge, however, there have been no studies comparing cortical atrophy according to the age group in AD continuum patients, which can be regarded as pathological aging, with physiological brain aging. Considering that aging is a strong risk factor for AD (Scheltens et al., 2016), attempts to differentiate age-related pathology from physiological brain aging at a very early stage are important for establishing new strategies to prevent accelerated pathological brain aging.

In this study, we therefore investigated the long-term trajectory of physiological and pathological brain aging in a large cohort comprising individuals ranging from the 50s to over 80 years of age. Our main objective was to explore specific brain regions in order to distinguish pathological brain aging from physiological brain aging using sophisticated measurements of cortical thickness. We hypothesized that there would be distinct brain regions between physiological and pathological brain aging. To address different stages of the AD continuum, we studied seven groups of participants according to the severity of cognitive impairment: CN; SMI; early-stage aMCI; late-stage aMCI; very mild AD; mild AD; and moderate to severe AD.

## Materials and Methods

### Study Participants

Cognitively normal individuals were recruited from the Health Promotion Center of the Samsung Medical Center (Seoul, South Korea). The study population comprised men and women aged 50 years or older who underwent a comprehensive health screening exam from January 1, 2009 to December 31, 2014. There were 3,290 eligible participants who attended a preventative medical check-up, which included an assessment of cognitive function and dementia status. All study participants underwent a high-resolution 3.0-Tesla brain MRI, including three-dimensional (3D) volume images, as a part of their dementia assessment. The assessment procedure used for the participants has been described in detail elsewhere (Lee et al., 2016). We excluded participants who had any of the following conditions: 202 participants with missing data on education years or Mini-Mental State Examination (MMSE) score; 178 participants with significant cognitive impairments defined as an MMSE scores below the 16th percentile in age-, sex-, and education-matched norms or through an interview conducted by a qualified neurologist; and 136 participants with unreliable analyses of cortical thickness due to head motion, blurring of the MRI, inadequate registration to a standardized stereotaxic space, misclassification of the tissue type, or inexact surface extraction. Therefore, the final sample size was 2,823 participants (1,427 men and 1,396 women).

In addition, a total of 3,619 AD continuum patients were recruited from the Memory Disorders Clinic of Samsung Medical Center from March 1, 2007 to December 31, 2013. Our recruitment from the memory clinic was focused on enriching the number of patients with clinical AD, subcortical vascular dementia, MCI, or SMI. However, only a small minority of the recruited group had frontotemporal lobar dementia, dementia with Lewy bodies, or other degenerative dementias. We selected 2,770 patients at the age of 50 or older who were clinically diagnosed with SMI, aMCI, or AD dementia. The clinical diagnosis was established at a multi-disciplinary conference applying standard research criteria for SMI, aMCI, and dementia syndromes. In detail, all of the SMI patients met the following criteria (Rabin et al., 2015): (1) subjective memory complaints by patients or caregivers; (2) no objective cognitive dysfunction as evidenced by scores from evaluations on any cognitive domains; and (3) not suffering dementia. Patients were diagnosed with aMCI using the Petersen criteria (Petersen, 2004) with the following modifications, which have been previously described in detail (Seo et al., 2009): (1) a subjective cognitive complaint by the patient or his/her caregiver; (2) normal Activities of Daily Living (ADL) score determined clinically and with the instrumental ADL scale; (3) an objective cognitive decline below the 16th percentile [−1.0 standard deviation (SD)] of age- and education-matched norms in at least one of four cognitive domains (language, visuospatial, memory, or frontal-executive function) on neuropsychological tests; and (4) absence of dementia. Patients with AD dementia fit the NINCDS-ADRDA criteria for probable AD (McKhann et al., 2011). In this study, we excluded patients who met the Diagnostic and Statistical Manual of Mental Disorders (Fourth Edition) criteria for psychotic or mood disorder, such as schizophrenia or major depressive disorder. All patients underwent a standardized diagnostic assessment protocol including a high-resolution 3.0-Tesla MRI for neurodegenerative and cerebrovascular diseases and detailed neuropsychological tests. We excluded 95 patients with an unreliable analysis of the cortical thickness due to head motion, blurring of the MRI, inadequate registration to a standardized stereotaxic space, misclassification of tissue type, or inexact surface extraction. Therefore, the final sample size of the AD continuum patients was 2,675 (874 with SMI, 954 with aMCI, and 847 with AD).

Laboratory tests were conducted in all patients to rule out other causes of cognitive impairment. These tests included complete blood count, vitamin B12, folate, metabolite profile, thyroid function tests, and syphilis serology. Apolipoprotein E (*APOE*) genotyping was performed in 658 (23.3%) of the 2,823 CN individuals, 700 (80.1%) of the 874 patients with SMI, 803 (84.2%) of the 954 patients with aMCI, and 639 (75.4%) of the 847 patients with AD dementia, respectively. All study participants were excluded if they had a cerebral, cerebellar, or brainstem infarction; hemorrhage; brain tumor; hydrocephalus; severe cerebral white matter hyperintensities (deep white matter ≥ 2.5 cm and caps or band ≥ 1.0 cm); or severe head trauma by personal history.

### Standard Protocol Approvals, Registrations, and Patient Consent

This study was approved by the Institutional Review Board at the Samsung Medical Center. In addition, all methods were carried out in accordance with the approved guidelines. A written informed consent was obtained from all participants prior to the study.

### Neuropsychological Assessments

All AD continuum patients underwent neuropsychological tests using a standardized neuropsychological battery (Ahn et al., 2010). This included tests for attention, language, praxis, elements of Gerstmann syndrome, visuoconstructive function, verbal and visual memory, and frontal/executive function. The series also included digit span tests (forward and backward); the Korean version of the Boston Naming Test; the Rey-Osterrieth Complex Figure Test (RCFT), which involves copying, immediate and 20-min delayed recall, and recognition; the Seoul Verbal Learning Test (SVLT), which includes three learning-free recall trials of 12 words, a 20-min delayed recall trial for these 12 items, and a recognition test; a phonemic and semantic Controlled Oral Word Association Test; and a Stroop Test, which involves word and color reading of 112 items during a 2-min period. Each score was converted into a standardized score (Z score) based on age-, sex, and education-adjusted norms (Ahn et al., 2010). Scores lower than −1.0 SD from the age-, sex-, and education-adjusted norms were considered abnormal. The results of neuropsychological tests in AD continuum patients are presented in Supplementary Table 1.

### Classification of aMCI and AD

To investigate pathological brain aging, we further classified patients with aMCI or AD according to the severity of cognitive impairment. In aMCI patients, memory function was considered abnormal when delayed recall scores on either the SVLT or RCFT were lower than −1.0 SD from the baseline memory test results (Ye et al., 2013). Patients with performances between −1.0 and −1.5 SD of the age-, sex-, and education-adjusted norms were diagnosed with early-stage aMCI (305 patients), while those with performances lower than −1.5 SD were classified as late-stage aMCI (649 patients). In AD patients, dementia severity was classified based on the clinical dementia rating (CDR) score (Morris et al., 2001): very mild AD (CDR 0.5, 282 patients), mild AD (CDR 1, 411 patients), and moderate to severe AD (CDR 2 and 3, 154 patients).

### Brain MRI Scans

All study participants underwent a 3D volumetric brain MRI scan. An Achieva 3.0-Tesla MRI scanner (Philips, Best, Netherlands) was used to acquire a 3D T1 Turbo Field Echo (TFE) MRI data using the following imaging parameters: sagittal slice thickness, 1.0 mm with 50% overlap; no gap; repetition time of 9.9 ms; echo time of 4.6 ms; flip angle of 8°; and matrix size of 240 × 240 pixels reconstructed to 480 × 480 over a field view of 240 mm.

### Cortical Thickness Measurements

T1-weighted MR images were automatically processed using the standard Montreal Neurological Institute image processing software (CIVET) to measure cortical thickness. This software has been well-validated and extensively described elsewhere including in aging/atrophied brain studies (Lerch and Evans, 2005; Singh et al., 2006). In summary, native MR images were first registered into a standardized stereotaxic space using an affine transformation (Collins et al., 1994). Non-uniformity artifacts were corrected using the N3 algorithm, and the registered and corrected volumes were classified as GM, white matter (WM), cerebrospinal fluid (CSF), and background using an artificial neural net classifier (Sled et al., 1998). The surfaces of the inner and outer cortices were automatically extracted by deforming a spherical mesh onto the gray/white boundary of each hemisphere using the Constrained Laplacian-Based Automated Segmentation with Proximities algorithm, which has also been well-validated and extensively described elsewhere (Kim J.S. et al., 2005).

Cortical thickness was calculated as the Euclidean distance between the linked vertices of the inner and outer surfaces after application of an inverse transformation matrix to the cortical surfaces and reconstructing them in the native space (Kim J.S. et al., 2005; Im et al., 2006). To control for brain size, we computed the intracranial volume (ICV) using classified tissue information and a skull mask acquired from the T1-weighted image (Smith, 2002). ICV was defined as the total volume of GM, WM, and CSF, with consideration of the voxel dimension. Classified GM, WM, CSF, and background within the mask were transformed back into the individual native space.

To compare the thicknesses of corresponding regions among the participants, the thicknesses were spatially registered on an unbiased iterative group template by matching the sulcal folding pattern using surface-based registration involving sphere-to-sphere warping (Lyttelton et al., 2007). For global and lobar regional analyses, we used the lobe-parcellated group template that had been previously divided into frontal, temporal, parietal, and occipital lobes using SUMA^1^ (Im et al., 2006). Average thickness values of the whole vertex in each hemisphere and lobar region were used for global analysis.

### Statistical Analysis

The Student’s *t*-test, Chi-square test, and analysis of variance with Bonferroni *post hoc* tests were used as appropriate to compare the demographic and clinical characteristics of the groups (diagnostic or age groups). To evaluate the relationship between age (continuous) and cortical thickness, we used multiple linear regression analysis after controlling for sex, education years (continuous), ICV, vascular risk factors (hypertension, diabetes mellitus, and hyperlipidemia), and history of ischemic heart disease or stroke.

For cortical thickness analyses of MRI data from CN individuals and AD continuum patients, we used a MATLAB-based toolbox (available free online at the University of Chicago website^2^). Diffusion smoothing with a full-width half-maximum of 20 mm was used to blur each cortical thickness map, leading to an increased signal-to-noise ratio and statistical power (Lerch and Evans, 2005). We entered age (continuous or categorical) as a predictor and vertex-by-vertex cortical thickness as an outcome to analyze the relationship between cortical thickness and age in the surface model. A linear regression analysis was then performed after controlling for sex, education years (continuous), ICV, vascular risk factors (hypertension, diabetes mellitus, and hyperlipidemia), and history of ischemic heart disease or stroke. The cortical surface model contained 81,924 vertices; thus, correction for multiple comparisons was performed using a random field theory correction at a probability value of 0.05. Statistical analyses were performed using SPSS version 20.0 (SPSS, Inc., Chicago, IL, United States).

## Results

### Characteristics of the Study Participants

Table 1 shows the demographic and clinical characteristics of the study participants. The mean (SD) ages of the CN, SMI, aMCI, and AD dementia group were 64.1 (6.9), 65.9 (8.5), 71.1 (8.4), and 73.0 (9.2) years, respectively. Significant differences in the mean age among the four groups were noted. The proportion of female was highest in the SMI group (72.2%), while it was lowest in the CN group (49.5%). The AD dementia group had the lowest education years and ICV, but the proportion of *APOE* ε4 carriers was highest among the four groups. The number of study participants in each group is presented in Supplementary Table 2. In the CN, aMCI, and AD dementia groups, we combined the age groups of the 90 and 100s into the over-80 groups due to the small number of participants in these age groups.

**TABLE 1:** Demographic and clinical characteristics of the study participants (*N* = 5,498).

				**aMCI**			**AD dementia**			
				
	**Total**	**CN**	**SMI**	**Total**	**Early-stage**	**Late-stage**	**Total**	**Very mild**	**Mild**	**Moderate to severe**
*N*	5,498	2,823	874	954	305	649	847	282	411	154
Age, years	67.0 (8.6)	64.1 (6.9)^a,b,c^	65.9 (8.5)^a,d,e^	71.1 (8.4)^b,d,f^	70.3 (8.2)	71.5 (8.5)	73.0 (9.2)^c,e,f^	72.5 (9.3)	73.0 (8.7)	73.9 (10.3)
Age range, years	50–101	50–91	50–89	50–99	50–88	50–99	50–101	50–101	51–91	50–91
Female	3,160 (57.5)	1,396 (49.5)^a,b,c^	631 (72.2)^a,d,e^	575 (60.3)^b,d,f^	200 (65.6)	375 (57.8)	558 (65.9)^c,e,f^	179 (63.5)	263 (64.0)	116 (75.3)
Education, years	11.6 (4.9)	12.7 (4.3)^a,b,c^	11.1 (5.0)^a,e^	10.5 (5.1)^b,f^	9.3 (5.8)	11.1 (4.6)	9.6 (5.5)^c,e,f^	9.6 (5.6)	9.8 (5.6)	9.3 (5.1)
*APOE* ε4 present^*^	890 (31.8)	124 (18.8)^b,c^	149 (21.3)^d,e^	290 (36.1)^b,d,f^	71 (28.3)	219 (39.7)	327 (51.2)^c,e,f^	125 (53.9)	152 (50.0)	50 (48.5)
MMSE, score	26.2 (4.5)	28.1 (1.7)^b,c^	28.3 (2.1)^d,e^	25.7 (3.4)^b,d,f^	26.0 (3.5)	25.5 (3.3)	18.4 (5.3)^c,e,f^	21.3 (3.9)	18.6 (4.2)	12.6 (5.5)
CDR, score	0.7 (0.5)	−	0.0 (0.0)	0.5 (0.1)^f^	0.5 (0.1)	0.5 (0.1)	1.0 (0.6)^f^	0.5 (0.0)	1.0 (0.0)	2.1 (0.3)
Hypertension	2,395 (43.6)	1,303 (46.2)^a,^	294 (33.6)^a,d,e^	425 (44.5)^d^	130 (42.6)	295 (45.5)	373 (44.0)^e^	130 (46.1)	175 (42.6)	68 (44.2)
DM	1,173 (21.3)	476 (16.9)^a,b,c^	183 (20.9)^a,d,e^	271 (28.4)^b,d^	78 (25.6)	193 (29.7)	243 (28.7)^c,e^	83 (29.4)	117 (28.5)	43 (27.9)
Hyperlipidemia	1,660 (30.2)	935 (33.1)^b,c^	273 (31.2)^e^	259 (27.1)^b,f^	78 (25.6)	181 (27.9)	193 (22.8)^c,e,f^	69 (24.5)	94 (22.9)	30 (19.5)
History of IHD	490 (8.9)	158 (5.6)^a,b,c^	119 (13.6)^a,e^	130 (13.6)^b,f^	45 (14.8)	85 (13.1)	83 (9.8)^c,e,f^	30 (10.6)	39 (9.5)	14 (9.1)
History of stroke	196 (3.6)	80 (2.8)^b,c^	23 (2.6)^d,e^	49 (5.1)^b,d^	16 (5.2)	33 (5.1)	44 (5.2)^c,e^	20 (7.1)	19 (4.6)	5 (3.2)
ICV (cm^3^)	1,332.9 (126.4)	1,358.4 (122.4)^a,b,c^	1,313.7 (121.9)^a,e^	1,311.5 (123.8)^b,f^	1,314.5 (126.0)	1,310.1 (122.7)	1,291.9 (128.5)^c,e,f^	1,295.4 (121.6)	1,293.2 (129.4)	1,282.0 (138.6)

Values are mean (SD) or N (%). ^*^APOE genotyping was performed in 658 (23.3%) of the 2,823 CN individuals, 700 (80.1%) of the 874 patients with SMI, 803 (84.2%) of the 954 patients with aMCI, and 639 (75.4%) of the 847 patients with AD dementia, respectively. ^a,b,c,d,e,f^Scores in each row are significantly different in the analysis of variance with Bonferroni’s corrections, Chi-square or Fisher’s exact tests: ^a^CN vs. SMI; ^b^CN vs. aMCI; ^c^CN vs. AD dementia; ^d^SMI vs. aMCI; ^e^SMI vs. AD dementia; ^f^aMCI vs. AD dementia. N, number; SD, standard deviation; CN, cognitively normal; SMI, subjective memory impairment; aMCI, amnestic mild cognitive impairment; AD, Alzheimer’s disease; *APOE*, apolipoprotein E; MMSE, Mini-Mental State Examination; CDR, clinical dementia rating; DM, diabetes mellitus; IHD, ischemic heart disease; ICV, intracranial volume.

### Mean Cortical Thickness of Each Age Group According to the Diagnostic Groups

The mean and SD of the cortical thickness for each age group according to the diagnostic groups is presented in Table 2. In the CN, SMI, and aMCI (early- and late-stages) groups, the mean cortical thickness in the global, frontal, temporal, parietal, and occipital regions decreased as the age increased. However, among patients with very mild and mild AD, the 50s group had a lower mean cortical thickness in the parietal and occipital regions than the 60 and 70s groups. Among patients with moderate to severe AD, the 50s group exhibited the lowest mean cortical thickness in the frontal, parietal, and occipital regions.

**TABLE 2:** Mean and SD of the cortical thickness for each age group according to the diagnostic groups.

**Age group**	***N***	**Global**	**Frontal**	**Temporal**	**Parietal**	**Occipital**
**CN**						
50s	694	3.08 (0.10)	3.13 (0.10)	3.23 (0.15)	2.95 (0.14)	2.72 (0.12)
60s	1,509	3.05 (0.10)	3.10 (0.11)	3.22 (0.15)	2.92 (0.13)	2.70 (0.12)
70s	572	2.99 (0.12)	3.04 (0.13)	3.16 (0.17)	2.85 (0.14)	2.63 (0.13)
Over-80	48	2.98 (0.16)	3.03 (0.16)	3.14 (0.19)	2.83 (0.17)	2.63 (0.16)
**SMI**						
50s	224	3.09 (0.09)	3.16 (0.10)	3.28 (0.10)	2.93 (0.10)	2.70 (0.11)
60s	348	3.04 (0.10)	3.11 (0.11)	3.24 (0.11)	2.89 (0.11)	2.65 (0.11)
70s	247	2.96 (0.12)	3.02 (0.13)	3.14 (0.15)	2.80 (0.13)	2.58 (0.13)
Over-80	55	2.91 (0.13)	2.98 (0.14)	3.06 (0.14)	2.77 (0.15)	2.54 (0.12)
**Early-stage aMCI**						
50s	35	3.11 (0.09)	3.18 (0.10)	3.30 (0.10)	2.96 (0.08)	2.73 (0.10)
60s	96	3.03 (0.10)	3.10 (0.11)	3.23 (0.11)	2.88 (0.11)	2.65 (0.12)
70s	137	2.92 (0.12)	2.98 (0.14)	3.08 (0.14)	2.78 (0.13)	2.56 (0.12)
Over-80	37	2.85 (0.14)	2.92 (0.15)	3.01 (0.16)	2.71 (0.15)	2.49 (0.14)
**Late-stage aMCI**						
50s	62	3.03 (0.13)	3.11 (0.14)	3.21 (0.16)	2.87 (0.13)	2.66 (0.12)
60s	193	2.98 (0.12)	3.05 (0.12)	3.15 (0.15)	2.82 (0.13)	2.61 (0.12)
70s	288	2.89 (0.13)	2.96 (0.14)	3.04 (0.16)	2.74 (0.13)	2.54 (0.13)
Over-80	106	2.82 (0.13)	2.89 (0.14)	2.95 (0.16)	2.68 (0.13)	2.49 (0.14)
**Very mild AD**						
50s	35	2.88 (0.15)	3.00 (0.13)	3.05 (0.18)	2.65 (0.19)	2.49 (0.20)
60s	56	2.90 (0.16)	2.99 (0.14)	3.04 (0.20)	2.74 (0.19)	2.53 (0.19)
70s	125	2.84 (0.14)	2.92 (0.15)	2.97 (0.17)	2.70 (0.15)	2.50 (0.14)
Over-80	66	2.77 (0.15)	2.85 (0.15)	2.89 (0.19)	2.65 (0.17)	2.45 (0.16)
**Mild AD**						
50s	43	2.78 (0.10)	2.91 (0.12)	2.91 (0.15)	2.55 (0.12)	2.41 (0.15)
60s	82	2.81 (0.14)	2.91 (0.13)	2.93 (0.18)	2.65 (0.15)	2.47 (0.17)
70s	198	2.81 (0.15)	2.90 (0.15)	2.91 (0.19)	2.69 (0.17)	2.50 (0.15)
Over-80	88	2.78 (0.13)	2.85 (0.13)	2.87 (0.17)	2.67 (0.15)	2.47 (0.15)
**Moderate to severe AD**						
50s	20	2.64 (0.16)	2.73 (0.20)	2.79 (0.22)	2.44 (0.13)	2.34 (0.18)
60s	29	2.62 (0.17)	2.74 (0.18)	2.69 (0.21)	2.48 (0.20)	2.32 (0.22)
70s	54	2.66 (0.17)	2.76 (0.17)	2.69 (0.22)	2.55 (0.17)	2.37 (0.18)
Over-80	51	2.71 (0.18)	2.79 (0.18)	2.76 (0.23)	2.62 (0.19)	2.41 (0.19)

*Values are N or mean cortical thickness (SD). N, number; SD, standard deviation; CN, cognitively normal; SMI, subjective memory impairment; aMCI, amnestic mild cognitive impairment; AD, Alzheimer’s disease.*

### Relationship Between Age and Cortical Thickness According to the Diagnostic Groups

**FIGURE 1:**
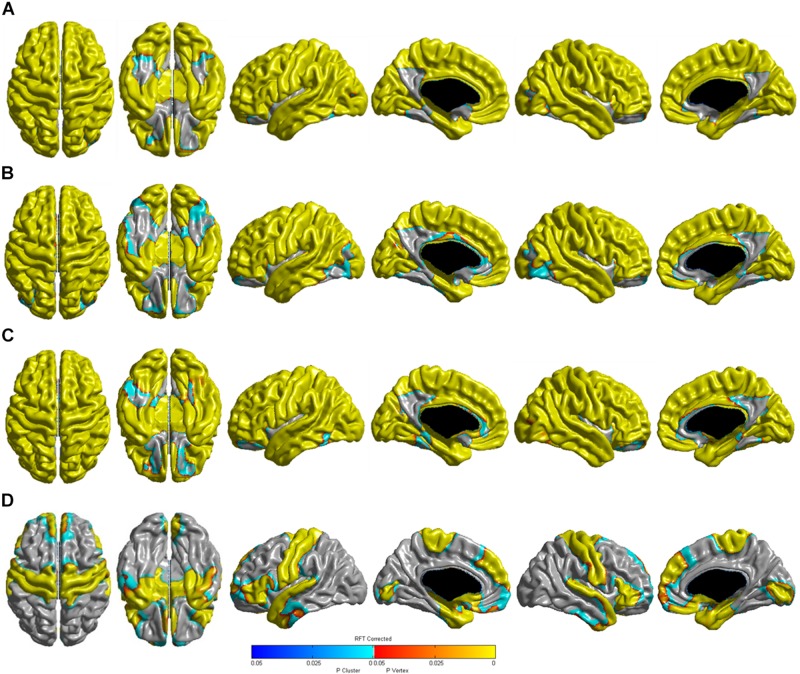
Three-dimensional reconstruction of the correlation between age (continuous) and cortical thickness in the **(A)** CN, **(B)** SMI, **(C)** aMCI, and **(D)** AD groups. RFT-corrected (*P*-value < 0.05) results were adjusted for sex, education years, ICV, vascular risk factors (hypertension, diabetes mellitus, and hyperlipidemia), and history of ischemic heart disease or stroke. The color scale from blue to yellow indicates statistically significant age-related cortical thinning. CN, cognitively normal; SMI, subjective memory impairment; aMCI, amnestic mild cognitive impairment; AD, Alzheimer’s disease; RFT, random field theory; ICV, intracranial volume.

Table 3 shows the relationships between age and cortical thickness according to the diagnostic groups. Multiple linear regression analyses showed that age was negatively correlated with cortical thickness in the global, frontal, temporal, parietal, and occipital regions in the CN, SMI, and aMCI (early- and late-stages) groups (all *P* < 0.001). In the very mild AD groups, age was negatively correlated with cortical thickness in the global (*P* < 0.001), frontal (*P* < 0.001), and temporal regions (*P* < 0.001), but not in the parietal (*P* = 0.079) and occipital (*P* = 0.061) regions. Age was positively correlated with cortical thickness in the parietal region in the mild AD (*P* = 0.001) and moderate to severe AD (*P* < 0.001) groups. The 3D reconstruction for the correlation between age and cortical thickness of the diagnostic groups is presented in Figure 1. The topography of age-related cortical thinning was widespread and severe in the CN, SMI, and aMCI groups. However, the precuneus and inferior temporal regions were relatively preserved against the effects of age (Figures 1A–C). In the AD group, increasing age was associated with cortical thinning in the medial and ventrolateral prefrontal, medial and lateral temporal, medial occipital, precentral, and post-central regions (Figure 1D).

**TABLE 3:** Relationship between age and cortical thickness according to the diagnostic groups.

	**Global**			**Frontal**			**Temporal**			**Parietal**			**Occipital**		
	
	***B***	**SE**	***P***	***B***	**SE**	***P***	***B***	**SE**	***P***	***B***	**SE**	***P***	***B***	**SE**	***P***
**CN**															
Age	–0.004	<0.001	<0.001	–0.004	<0.001	<0.001	–0.004	<0.001	<0.001	–0.005	<0.001	<0.001	–0.005	<0.001	<0.001
**SMI**															
Age	–0.006	<0.001	<0.001	–0.006	<0.001	<0.001	–0.007	0.001	<0.001	–0.005	<0.001	<0.001	–0.006	<0.001	<0.001
**Early-stage aMCI**															
Age	–0.009	0.001	<0.001	–0.009	0.001	<0.001	–0.012	0.001	<0.001	–0.009	0.001	<0.001	–0.008	0.001	<0.001
**Late-stage aMCI**															
Age	–0.008	0.001	<0.001	–0.008	0.001	<0.001	–0.010	0.001	<0.001	–0.007	0.001	<0.001	–0.006	0.001	<0.001
**Very mild AD**															
Age	–0.005	0.001	<0.001	–0.006	0.001	<0.001	–0.007	0.001	<0.001	–0.002	0.001	0.079	–0.002	0.001	0.061
**Mild AD**															
Age	–0.001	0.001	0.416	–0.002	0.001	0.009	–0.003	0.001	0.012	0.003	0.001	0.001	0.002	0.001	0.095
**Moderate to severe AD**															
Age	0.002	0.001	0.179	0.001	0.001	0.436	<0.001	0.002	0.827	0.006	0.001	<0.001	0.002	0.002	0.193

**B* (SE): β value (standard error of the mean). Multiple linear regression analysis was performed after controlling for sex, education years (continuous), ICV, vascular risk factors (hypertension, diabetes mellitus, and hyperlipidemia), and history of ischemic heart disease or stroke. CN, cognitively normal; SMI, subjective memory impairment; aMCI, amnestic mild cognitive impairment; AD, Alzheimer’s disease.*

### Topographical Differences in Cortical Thickness of Each Age Group Based on the Diagnostic Groups Compared to Age Group-Matched CN Individuals

**FIGURE 2:**
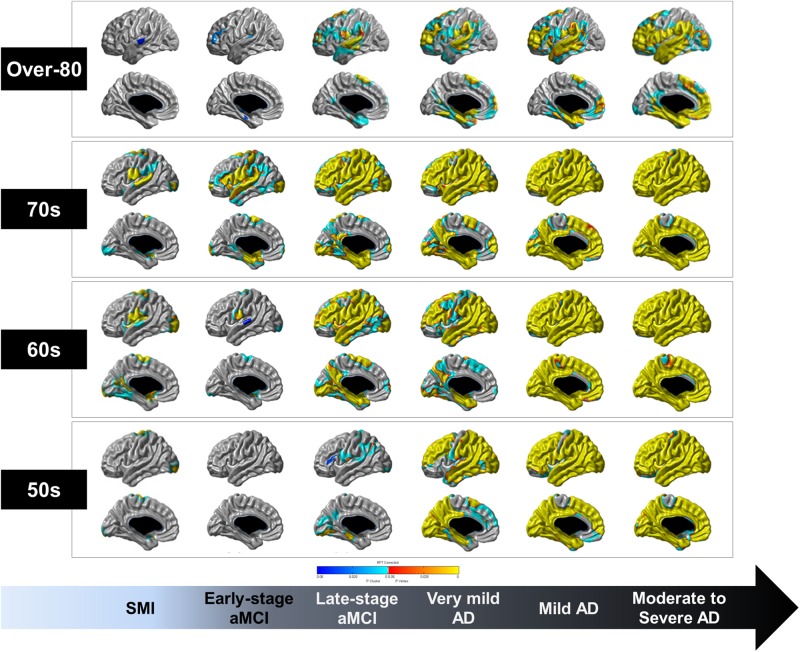
Topographical differences in the cortical thickness of each age group based on the diagnostic groups compared to age group-matched CN individuals. RFT-corrected (*P*-value < 0.05) results were adjusted for sex, education years, ICV, vascular risk factors (hypertension, diabetes mellitus, and hyperlipidemia), and history of ischemic heart disease or stroke. The color scale from blue to yellow indicates statistically significant topographical differences in cortical thinning of each age group. CN, cognitively normal; SMI, subjective memory impairment; aMCI, amnestic mild cognitive impairment; AD, Alzheimer’s disease; RFT, random field theory; ICV, intracranial volume.

Figure 2 shows the topographical differences in cortical thickness of each age group based on the diagnostic groups compared to the age-matched CN group. From the SMI to moderate to severe AD stages, cortical thinning occurred in the perisylvian region and spread widely in the 60 and 70s groups. Notably, the precuneus and inferior temporal regions, which were relatively preserved against age-related cortical thinning, were especially affected at the late-stage aMCI stage and cortical thinning occurred across most of the cortices in the moderate to severe AD stage. However, cortical thinning of the paracentral lobule and anterior cingulate regions were relatively less affected until later in life, even at the stage of moderate to severe AD. Compared to the over-80 CN group, the over-80 late-stage aMCI group showed cortical thinning in the dorsolateral prefrontal, perisylvian, and medial temporal regions. However, the precuneus, paracentral lobule, anterior cingulate, medial occipital regions were relatively preserved until the moderate to severe AD stage.

## Discussion

In this study, we investigated the trajectory of physiological and pathological brain aging in a large population of 5,498 participants using sophisticated measurements of cortical thickness. The major findings of the study were as follows. First, aging extensively affected cortical thinning not only in CN individuals but also in AD continuum patients; however, the precuneus and inferior temporal regions were relatively preserved against age-related cortical thinning. Second, compared to CN individuals, AD continuum patients showed a decreased cortical thickness in the perisylvian region even in the SMI stage. However, widespread cortical thinning including the precuneus and inferior temporal regions were found in late-stage aMCI and moderate to severe AD. Third, unlike the other age groups, the over-80 AD continuum patients showed prominent cortical thinning in the medial temporal region with relative sparing of the precuneus. Taken together, our findings provide some important insights into the difference between physiological and pathological brain aging.

Age-related cortical thinning was widespread and severe in the CN, SMI, and aMCI groups, whereas in the AD group, increasing age was not associated with cortical thinning in the dorsolateral prefrontal, parietal, and lateral occipital regions. Besides, age was positively correlated with mean cortical thickness in the parietal region in the mild AD and moderate to severe AD groups. In AD patients, the age of symptom onset is known to determine distinctive radiologic features (Kim E.J. et al., 2005; Cho et al., 2013); specifically, patients with early-onset AD have greater cortical atrophy in the lateral parietal region and precuneus than those with late-onset AD (Frisoni et al., 2007; Ossenkoppele et al., 2015a). This could be explained by the brain reserve hypothesis (Stern, 2002), which postulates that early-onset AD have a greater brain reserve than late-onset AD and therefore more severe imaging abnormalities despite having similar cognitive performance. We therefore suggest that early-onset AD is more severely affected by pathological brain aging than physiological brain aging.

To evaluate pathological brain aging more specifically, we classified the AD continuum patients into six groups according to the severity of cognitive worsening. Through comparisons with CN individuals in each age group, we minimalized the effects of physiological aging and evaluated the distinct brain regions associated with physiological and pathological brain aging. Cortical thinning in the perisylvian region was found from the SMI group and spread widely as pathological brain aging progressed. Previous studies have shown that SMI patients, compared to CN individuals, had AD-like cortical atrophy patterns (Peter et al., 2014; Meiberth et al., 2015; Schultz et al., 2015). However, some studies did not find significant differences between SMI patients and CN individuals (Selnes et al., 2012; Jung et al., 2016). This disparity is probably due to the fact that SMI is a heterogeneous group that includes preclinical AD or various conditions that can affect cognition, such as anxiety and depression (Jessen et al., 2014). Nonetheless, we found that there was a distinctive cortical thinning region – the perisylvian area – between the SMI and CN groups with a large sample size. The perisylvian area has been linked to the lateral cholinergic pathway (Selden et al., 1998). Considering that the cholinergic system plays an important role in learning and memory (Hasselmo, 2006), our findings may provide clues for future research on SMI, as a part of AD continuum.

Another noteworthy finding was that widespread cortical thinning including the precuneus and inferior temporal regions were found from the late-stage aMCI to the moderate to severe AD. As mentioned above, the precuneus and inferior temporal regions were relatively preserved against the effects of aging in the CN, SMI, and aMCI groups; however, these regions exhibited cortical thinning that clearly distinguished between the early- and late-stage aMCI. The precuneus has been reported to have a central role in highly integrated tasks, including episodic memory retrieval, visuospatial processing, and self-consciousness (Cavanna and Trimble, 2006). The current literature suggests that the precuneus is particularly vulnerable to the early deposition of β-amyloid (Sperling et al., 2009) and seems to be affected even in the early-stages of AD (Greicius et al., 2004; Klunk et al., 2004; Miners et al., 2016). In addition, the inferior temporal region is considered particularly important for the ventral stream and has been implicated in object recognition and semantic processing (Grill-Spector et al., 2000; Kellenbach et al., 2005). Previous studies also demonstrated that the inferior temporal region is affected during the prodromal stage of AD and may underlie some of the early AD-related clinical dysfunctions (Convit et al., 2000; Scheff et al., 2011). We therefore suggest that the precuneus and inferior temporal regions are key regions in distinguishing between physiological and pathological brain aging, although cortical thinning is initiated in the perisylvian region, even at the SMI stage.

Interestingly, unlike the other age groups, the over-80 AD continuum patients showed minimal cortical thinning in the perisylvian region from SMI; however, prominent cortical thinning was found in the medial temporal region with relative sparing of the precuneus. This might be due to the specific pattern of cortical thinning that occurs in late-onset AD. In fact, previous studies from our group showed that patients with late-onset AD presented cortical thinning mostly in the medial temporal region, while early-onset AD patients had prominent cortical thinning in the precuneus (Seo et al., 2011; Cho et al., 2013). Alternatively, considering 25–50% of the oldest-old have “low” or “none” neuritic amyloid plaque density (Neltner et al., 2016), this may be partly explained by suspected non-Alzheimer pathology or primary age-related tauopathy (PART). As aging progresses, burdens of neurodegenerative and cerebrovascular diseases increase (Braak et al., 2011; Thal et al., 2012). Moreover, a meta-analysis showed that the frequency of amyloid positivity actually decreased with aging in patients with clinical AD dementia (Ossenkoppele et al., 2015b). Recent studies demonstrated that the oldest-old patients with PART showed significantly less extensive tau lesions beyond the medial temporal lobe differing from those in AD (Jellinger, 2018; Bell et al., 2019). As a result, the oldest-old patients may be clinically misdiagnosed with AD. Further studies are needed to determine the underlying pathology in the oldest-old patients with clinical AD dementia.

The strengths of the study include the large number of participants (*N* = 5,498) and the sophisticated measurements of cortical thickness using the same type of scanner with the same scan parameters across different waves of data collection. However, several limitations should be acknowledged when interpreting the results. First, our study was designed to be cross-sectional, precluding claims of causality. The cross-sectional design did not take into account individual differences in the process of aging. Second, in the present study, CN individuals were recruited from individuals seeking a comprehensive preventive health exam not covered by national medical insurance, which might not be completely representative of the general population. Third, we did not have additional biomarkers indicating AD pathology, such as CSF biomarkers, molecular imaging or neuropathological data from the participants. Finally, despite the large sample size, some subgroups such as the 50s and over-80 groups in early-stage aMCI or the 50s group in very mild AD, were relatively small.

Nevertheless, our findings provide an important clue to understanding the mechanism of brain aging. Early identification of age-related pathology in physiological brain aging may be important to establish new strategies for preventing accelerated pathological brain aging, in keeping with the paradigm shift in focus from AD dementia to preclinical AD in the development of therapeutic interventions.

## Data Availability

The datasets generated for this study are available on request to the corresponding author.

## Ethics Statement

This study was approved by the Institutional Review Board at the Samsung Medical Center. In addition, all methods were carried out in accordance with the approved guidelines. A written informed consent was obtained from all participants prior to the study.

## Author Contributions

JL and SS conceived and designed the study, and drafted and revised the manuscript. JL, SC, SK, JK, YJ, HK, HJ, DN, and SS acquired the data. JL, YP, SP, UY, YC, BC, AH, SC, SK, JK, YJ, K-CP, HK, HJ, DN, and SS analyzed and interpreted the data. SS approved the final manuscript.

## Conflict of Interest Statement

The authors declare that the research was conducted in the absence of any commercial or financial relationships that could be construed as a potential conflict of interest.

## Footnotes

1 http://afni.nimh.nih.gov

2 http://galton.uchicago.edu/faculty/InMemoriam/worsley/research/surfstat/
