# Supplementary material for: Distinct Brain Regions in Physiological and Pathological Brain Aging
Source: Front Aging Neurosci. 2019 Jun 18;11:147. doi: 10.3389/fnagi.2019.00147 (PMC6591468; doi:10.3389/fnagi.2019.00147)
Supplement: Supplementary file 1 [file Table_1.docx]

**Distinct Brain Regions in Physiological and**

**Pathological Brain Aging**

Jin San Lee,^1-3^ Yu Hyun Park,^1,2^ Seongbeom Park,^1,2^ Uicheul Yoon,^4^ Yeongsim Choe,^1,2^ Bo Kyoung Cheon,^1,2^ Alice Hahn,^1,2^ Soo Hyun Cho,^5^ Seung Joo Kim,^6^ Jun Pyo Kim,^1,2^ Young Hee Jung,^1,2^ Key-Chung Park,^3^ Hee Jin Kim,^1,2^ Hyemin Jang,^1,2^ Duk L. Na,^1,2^ and Sang Won Seo^1,2,7,8*^

^1^Department of Neurology, Samsung Medical Center, Sungkyunkwan University School of Medicine, Seoul, South Korea

^2^Neuroscience Center, Samsung Medical Center, Seoul, South Korea

^3^Department of Neurology, Kyung Hee University Hospital, Seoul, South Korea

^4^Department of Biomedical Engineering, Daegu Catholic University, Gyeongsan, South Korea

^5^Department of Neurology, Chonnam National University Medical School, Gwangju, South Korea

^6^Department of Neurology, Gyeongsang National University School of Medicine and Gyeongsang National University Changwon Hospital, Changwon, South Korea

^7^Samsung Alzheimer Research Center, Center for Clinical Epidemiology, Samsung Medical Center, Seoul, South Korea

^8^Department of Health Sciences and Technology, Clinical Research Design and Evaluation, SAIHST, Sungkyunkwan University, Seoul, South Korea

***Correspondence:** Sang Won Seo, MD, PhD

Department of Neurology, Samsung Medical Center, Sungkyunkwan University School of Medicine, 81 Irwon-ro, Kangnam-ku, Seoul 06351, South Korea

Tel: +82-2-3410-1233, Fax: +82-2-3410-0052, E-mail address: sangwonseo@empal.com

**Running Title:** Physiological and pathological brain aging

**Supplementary data:** Supplementary Table 1 and 2

**Supplementary Table 1** Neuropsychological performance in AD continuum patients

| **Neuropsychological tests** | **SMI** | **aMCI** | | | **AD dementia** | | | |
| --- | --- | --- | --- | --- | --- | --- | --- | --- |
|  |  | **Total** | **Early-stage** | **Late-stage** | **Total** | **Very mild** | **Mild** | **Moderate to severe** |
| **Digit span: Forward** | 0.4 ± 1.1 | -0.1 ± 1.1 | -0.1 ± 1.1 | -0.1 ± 1.1 | -0.5 ± 1.2 | -0.3 ± 1.1 | -0.5 ± 1.1 | -0.9 ± 1.5 |
| **Digit span: Backward** | 0.4 ± 1.3 | -0.3 ± 1.2 | -0.3 ± 1.3 | -0.3 ± 1.1 | -1.0 ± 1.6 | -0.7 ± 1.3 | -1.0 ± 1.5 | -1.9 ± 2.2 |
| **K-BNT** | 0.6 ± 4.8 | -0.6 ± 1.7 | -0.3 ± 1.1 | -0.7 ± 1.9 | -2.9 ± 5.3 | -1.7 ± 3.3 | -2.7 ± 5.7 | -5.9 ± 6.3 |
| **RCFT: Copy** | 0.7 ± 1.1 | -0.8 ± 1.1 | -0.3 ± 1.1 | -1.0 ± 1.1 | -1.8 ± 1.3 | -1.3 ± 1.2 | -1.8 ± 1.3 | -2.6 ± 1.4 |
| **SVLT: Immediate recall** | 0.5 ± 1.0 | -1.6 ± 1.4 | -0.7 ± 0.9 | -2.1 ± 1.4 | -2.6 ± 1.5 | -2.4 ± 1.5 | -2.6 ± 1.5 | -2.9 ± 1.7 |
| **SVLT: Delayed recall** | 0.4 ± 0.8 | -0.9 ± 1.2 | -0.3 ± 1.0 | -1.1 ± 1.3 | -2.1 ± 1.5 | -1.8 ± 1.5 | -2.2 ± 1.4 | -2.7 ± 1.6 |
| **SVLT: Recognition score** | 0.4 ± 0.9 | -1.3 ± 1.0 | -0.8 ± 0.8 | -1.5 ± 1.0 | -2.0 ± 0.9 | -1.8 ± 1.0 | -2.1 ± 0.9 | -2.3 ± 0.9 |
| **RCFT: Immediate recall** | 0.3 ± 0.8 | -1.3 ± 0.9 | -0.9 ± 0.7 | -1.6 ± 0.9 | -2.1 ± 0.8 | -1.9 ± 0.8 | -2.2 ± 0.7 | -2.3 ± 0.7 |
| **RCFT: Delayed recall** | 0.6 ± 1.2 | -0.6 ± 1.8 | -0.2 ± 1.3 | -0.8 ± 2.0 | -2.5 ± 2.6 | -1.9 ± 2.1 | -2.6 ± 2.5 | -3.7 ± 3.5 |
| **RCFT: Recognition score** | 0.2 ± 1.1 | -0.8 ± 1.1 | -0.6 ± 1.1 | -0.9 ± 1.1 | -1.8 ± 1.2 | -1.3 ± 1.0 | -1.9 ± 1.0 | -2.7 ± 1.3 |
| **COWAT: Animal** | 0.3 ± 1.0 | -0.6 ± 1.0 | -0.4 ± 1.0 | -0.7 ± 1.1 | -1.6 ± 1.0 | -1.1 ± 1.0 | -1.7 ± 0.8 | -2.3 ± 1.0 |
| **COWAT: Supermarket** | 0.5 ± 1.2 | -0.3 ± 1.1 | -0.2 ± 1.0 | -0.4 ± 1.1 | -1.0 ± 1.0 | -0.6 ± 1.0 | -1.0 ± 1.0 | -1.6 ± 0.9 |
| **COWAT: Phonemic total** | 0.3 ± 1.0 | -0.9 ± 2.8 | -0.4 ± 4.4 | -1.1 ± 1.4 | -2.3 ± 1.8 | -1.8 ± 1.6 | -2.5 ± 1.8 | -3.1 ± 1.8 |
| **Stroop color reading** | 0.4 ± 1.1 | -0.1 ± 1.1 | -0.1 ± 1.1 | -0.1 ± 1.1 | -0.5 ± 1.2 | -0.3 ± 1.1 | -0.5 ± 1.1 | -0.9 ± 1.5 |

Values (Z-scores) are mean ± SD.

Abbreviations: AD = Alzheimer’s disease; SMI = subjective memory impairment; aMCI = amnestic mild cognitive impairment; K-BNT = Korean version of the Boston Naming Test; RCFT = Rey-Osterrieth Complex Figure Test; SVLT = Seoul Verbal Learning Test; COWAT = Controlled Oral Word Association Test; SD = standard deviation.

**Supplementary Table 2** Number of the study participants (N = 5,498)

|  | **CN** | **SMI** | **aMCI** | | | **AD dementia** | | | |
| --- | --- | --- | --- | --- | --- | --- | --- | --- | --- |
|  |  |  | **Total** | **Early-stage** | **Late-stage** | **Total** | **Very mild** | **Mild** | **Moderate to severe** |
| **Total** | 2823 | 874 | 954 | 305 | 649 | 847 | 282 | 411 | 154 |
| **50s** | 694 | 224 | 97 | 35 | 62 | 98 | 35 | 43 | 20 |
| **60s** | 1509 | 348 | 289 | 96 | 193 | 167 | 56 | 82 | 29 |
| **70s** | 572 | 247 | 425 | 137 | 288 | 377 | 125 | 198 | 54 |
| **80s** | 47 | 55 | 135 | 37 | 98 | 196 | 63 | 84 | 49 |
| **90s** | 1 |  | 8 |  | 8 | 8 | 2 | 4 | 2 |
| **100s** |  |  |  |  |  | 1 | 1 |  |  |

Abbreviations: CN = cognitively normal; SMI = subjective memory impairment; aMCI = amnestic mild cognitive impairment; AD = Alzheimer’s disease.
